# Transparency of Financial Reporting on Greenhouse Gas Emission Allowances: The Influence of Regulation

**DOI:** 10.3390/ijerph17030893

**Published:** 2020-01-31

**Authors:** Patricia Milanés Montero, Esteban Pérez Calderón, Ana Isabel Lourenço Dias

**Affiliations:** 1 Department of Accounting and Finance, University of Extremadura, Faculty of Economic and Business Sciences, 06006 Badajoz, Spain; estperez@unex.es; 2 Department of Financial Accounting, Lisbon Polytechnic Institute/Lisbon Accounting and Business School, 1069-035 Lisbon, Portugal

**Keywords:** EU ETS, Emission allowances, Greenhouse gas emissions, Transparency, Accounting regulation

## Abstract

This study focuses on the transparency of financial reporting on emission allowances (EA) and greenhouse gas (GHG) emissions within the European Union Emissions Trading Scheme (EU ETS). In particular, the different accounting treatments adopted by standard setters and professionals were analyzed to evaluate the influence of regulation in the transparency of financial reporting on EA and GHG emissions. Based on a sample of 85 companies registered with the Portuguese, Spanish, and French National Plans of Allocation (NPAs), data collected from the annual reports were analyzed for the 2008–2014 period. The results were obtained based on descriptive, logistic regressions and panel data statistical techniques, and they show that better levels of transparency of financial reporting on EA and GHG emissions are conditioned by a variety of accounting policies, which compromises the comparability of the financial information. The adoption of the International Accounting Standards Board (IASB) standards set lead to a greater dispersion in the choice of the accounting approach and a higher probability of not disclosing any information, as well as adopting off-balance sheet policies. Therefore, the regulatory factor is a determinant of the level of transparency of financial reporting on EA and GHG emissions, contributing to reduce strategies of omission.

## 1. Introduction

In the context of the EU ETS, a cap-and-trade scheme, the debate in the literature began on whether the company’s exposure generated an accounting treatment for EA and GHG emissions [1,2]. The basis for the functioning of the EU ETS, until the end of the year 2012 and with some application in phase III, was the grandfathering system. This system incorporates the prediction of the tons of GHG emissions necessary to an entity, so that it may continue with its operational activities and, at the same time, comply with the commitment of reduction of GHG emissions. This would result in the free allocation of that number of EA, decided by national governments on a National Plan of Allocation (NPA). At the end of a given period, the entities have to surrender EA equivalent to tons of emitted GHG. In the case of emitting GHG that exceed the cap, they have to acquire the corresponding EA that is missing for compliance; if the GHG emissions are below the cap, the surplus can be sold. Further discussion in the literature agrees that these transactions should be included in financial reporting [3,4,5,6,7,8,9], as they are relevant, in nature and value. However, the history of the regulation of financial reporting regarding EA and GHG emissions has been marked by the consecutive postponements of the IASB that is still without an answer for an explicit treatment. This lack of international guidance has been subjected to extensive criticism in the literature, and it is viewed as the main reason to the omission of accounting information on EA and GHG emissions in the financial reports [10,11,12,13,14,15]. Nevertheless, the full International Financial Reporting Standards (IFRS) presents general principles that may be applied. Back in 2004, the International Financial Reporting Interpretations Committee (IFRIC) elaborated Interpretation 3 ‘Emission Rights’, in accordance with the IASB Conceptual Framework (not reviewed at the date) and within the scope of International Accounting Standard (IAS) 38, IAS 20 and IAS 37. The absence of general support for IFRIC 3 led to its consequent withdrawal by the IASB, and allowed companies to base its financial reporting on the professional judgment—under the principles of IAS 8. As a reaction to the IASB position, some European national standard setters engaged on domestic solutions. Among others, it was the case of the Portuguese, Spanish and French standard setters: Comissão de Normalização Contabilística (CNC), Instituto de Contabilidad y Auditoría de Cuentas (ICAC) and Autorité des Normes Comptables (ANC), respectively. This scenario of duality between the international and domestic standard setters created some discrepancies in the adopted accounting treatments. Previous literature [3,11,12,13,14,15,16] identified different accounting policies, and even, the absence of disclosure. The purpose of the present study focuses that those practices may be compromising the transparency of financial reporting on EA and GHG emissions (not to discuss which accounting treatment is more suitable). The concept of transparency is not well defined in the literature, but it was understood as Barth and Schipper’s [17] state “the extent to which financial reports reveal an entity’s underlying economics in a way that is readily understandable by those using the financial reports.” The main purpose of the study is to reinforce the necessity of regulation to increase transparency of the financial reporting on the EU ETS transactions within IFRS with two arguments: IFRS companies are allowed not to disclose information on real transactions that have a regulated market behind (EA) and a position by the IASB could decrease the differences that national regulators apply in their issued standards. This study is an additional contribution to prior empirical studies in the area with particular emphasis on the transparency purpose of IFRS Foundation which we believe it is a weak position for the EU ETS transactions. The IFRS Foundation [18] state “Our mission is to develop IFRS Standards that bring transparency…”, although it is not explicit in the IASB Conceptual Framework that transparency is a purpose of the set of financial statements. In fact, IFRS companies may recognize and measure those transactions resorting IAS 38, or IFRS 9, for instance. So, although not completely disregarding the IFRS standards, there may be an omission of important financial information. The IASB position seems to be contrary to other regulators, such as European national regulators. This stronger position may contribute to increase the transparency of the financial reporting of the companies that are EU ETS participants. Therefore, this research distinguishes itself from previous studies because: (1) addresses the importance of transparency of financial reporting on EA and GHG emissions, and; (2) compares two scenarios of transparency within guidelines based on the (in)existence of specific accounting principles (domestic standards *versus* IFRS).

The selection of the sample was not made taking into consideration the level of pollution in each country. Instead of that, we selected three countries with interesting characteristics to study. For instance, the Portuguese companies showed a diversity of accounting policies both in terms of recognition and measurement of the EA and GHG emissions, while Spanish companies were more consensual on the recognition and more discordant on measurement. We believe that this is the consequence of the change in Portuguese legislation in 2010 (while Spanish Resolution of 2006 was maintained until 2016), as well as a more sense of compliance with environmental financial reporting regulation. These results (by country) were an initial justification for the role of regulation in the transparency of financial reporting. Therefore, the sample selection consists in 85 French, Spanish, and Portuguese companies listed in the NPAs of phase II and phase III, that have European operators exclusively in those three countries. This limitation was achieved by crossing the list of companies with available annual accounts with the operators listed in the report released by the European Commission on Verified Emissions for 2014 [19], and aggregate it to company-level. The individual financial statements were the first choice for a primary source. When not available, the information was collected from the consolidated financial statements. In these cases, an analysis of the perimeter of consolidation was the method to limit the sample to companies located in the three countries. Another condition to define the sample was the online availability of the financial reporting available in English, Spanish, Portuguese or French. The period of the study, from 2008 to 2014, represents the five years from phase II and the two initial years of phase III of the EU ETS. The implementation of the auction system was not seen as a major difference in the transparency of financial reporting as the surplus of EA back-loaded the distribution by auction [20]. After applying descriptive, logistic regression and panel data techniques, the results show that the transparency of financial reporting on EA and GHG emissions are negatively affected by the IFRS. Although the difference is not significantly different, there is more disparity when the IFRS are the basis of presentation.

This study is structured into five additional sections: In Section 2, we briefly present the literature review that enable the formulation of the hypothesis and describe the research method; the results are presented and discussed in Section 3, and the final considerations are presented in Section 4.

## 2. Materials and Methods

### 2.1. Development of the Hypothesis

#### 2.1.1. Financial Reporting on EA and GHG Emissions—An Overview

Back in 2005, the IASB released IFRIC 3 ‘Emission Rights’ for application after the beginning of phase I of the EU ETS, just to six months later decided to withdraw the interpretation arguing that there had been a misinterpretation of its urgency. In line with EFRAG’s negative advice, the IASB stated that the prescribed accounting treatment created mismatches in the financial statements. This was mainly due to the measurement discordant between the assets (the EA) and the liabilities (GHG emissions), that would result in artificial volatility of reported earnings and would not reflect the economic reality of the companies. The skepticism about the usefulness of the recognition of EA and GHG emissions [2,21] stemmed in financial reporting relatively neglected [22]. Previous descriptive studies [3,11,12,13,14,15], identified a multitude of accounting practices for the EU ETS transactions. Those can be distinguished into two basic approaches: Gross approaches (as prescribed by the IFRIC 3 or government grant approach identified by Ernst and Young [23], and net approaches (as prescribed by U.S. Generally Accepted Accounting Principles (GAAP). The U.S.A. regulation on EA is based on pronouncements issued by the Federal Energy Regulatory Commission (FERC)—Order 552 issued in 1993, codified in Uniform System of Accounts (USofA) 101.21. The prescribed accounting treatment may be summed in the following: (a) EA are not reflected in financial statements; (b) an asset is recorded as inventory for purchased emission certificates at its cost; (c) when an entity does not hold the estimated required amount of emission certificates, a liability is recognized to reflect the number and current price of lacking emission certificates. Those studies also identified relative high frequencies of non-disclosure that were associated with the IFRS lack of specific guidance. Giner [8] illustrates that although the financial reporting regarding EA and GHG emissions is not easily framed in conventional accounting categories, relying on the business model allows more discretion than imposing a common solution.

The role of the domestic standard setters was precisely to overcome the lack of international guidance and to find common ground for the accounting treatment regarding EA and GHG emissions. This was the case of: OIC—Organismo Italiano di Contabilità, DRSC—Deutsches Rechnungslegungs Standards Committee e.V.; AFRAG—Austrian Financial Reporting and Auditing Committee, DASB—Dutch Accounting Standards Board, CNC—Comissão de Normalização Contabilística, ICAC—Instituto de Contabilidad y Auditoría de Cuentas, ANC—Autorité des Normes Comptables. This study focuses on Portuguese, Spanish and French companies, and therefore it is presented in Table 1 a summary of the treatments prescribed by each national standard setter and by IFRIC 3.

#### 2.1.2. Transparency of Financial Reporting on EA and GHG Emissions and the Role of Accounting Regulation

One of the issues of introducing information on EA and GHG emissions in financial reporting has been the interpretation of the transactions from a market based on a cap-and-trade system, as materially relevant [24]. This is much due to the grandfathering system considered for allocation of EA in phases I, II and even phase III of the EU ETS. The surplus of EA from phase II amounted to around 2 billion EA at the beginning of phase III and increased to more than 2.1 billion in 2013. Consequently, the European Commission postponed the auction of 900 million EA until 2019-2020 [20]. Thus, to underline that information on EA and GHG emissions should be included in annual accounts, thus improving transparency, it is important to frame it within the concepts of relevance and faithful representation. The IASB Conceptual Frameworks (2010, 2018) expose them as qualitative characteristics. The concept of materiality is an entity-specific aspect of relevance based on nature or magnitude, or both, of the items to which the information relates in the context of an individual entity’s financial report (Conceptual Frameworks, 2010, 2018). As such, relevance is not limited to value relevance; it includes nature relevance, which should cover the specific nature of EA and GHG emissions under the EU ETS. The concept of faithful representation, states that financial reports represent economic phenomena in words and numbers, in a way that (i)t would be complete, neutral and free from error (Conceptual Frameworks, 2010, 2018). These qualities should be maximized to the extent possible in financial reporting. This clarification provides the basis to include information on EA and GHG emissions in financial reporting, as the practice of non-disclosure under the scope of materiality issues, is not under the complete concept of relevance nor under faithful representation.

Transparency is not a principle established in the Conceptual Frameworks. Nevertheless, the concept of financial reporting transparency, as exposed by Barth and Schipper [17], includes the perspective of the underlying economics, closely related to faithful representation: (…) [T]he extent to which financial reports reveal an entity’s underlying economics in a way that is readily understandable by those using the financial reports. Interestingly, Lovell and Mackenzie [25] argue that with the IASB-FASB (Financial Accounting Standards Board) joint project on ETS, accountants of major companies-participants in the EU ETS have suggested a readiness for clear guidance from the standard setters, due to a strong desire of reducing choice and be fairly compared with their competitors. The outcome of the joint project was not as expected, and in December of 2012 the IASB reactivated this project as an IASB-only research project. In 2015 the project was renamed to Pollutant Price Mechanisms, to reflect a change in its scope, approach and direction and it is seen as a research pipeline project, waiting for the revised Conceptual Framework is closed to finalization. Hence, it is announced that by 2019/2020, the Board will recommence the work [26]. Lovell et al. [11] and Giner [8] underline the importance of specific accounting treatment for obtaining a fair and transparent comparison of the financial reporting of EA and GHG emissions. However, this relation between specific regulation and increased transparency of financial reporting is not supported in the literature. In fact, such topics related to environmental disclosures are related to strategies of disclosure even in the presence of mandatory disclosure [27]. Moreover, IFRS provide several answers to include EU ETS transactions in the financial statements that do not include its omission (within IAS 38, IAS 20, IAS 37 or IFRS 9). Nevertheless, as some IFRS reporting companies choose to report some type of information on the exposure to the EU ETS, we believe that regulation would increase the levels of transparency and particularly, the possibility of comparison of the accounting policies between companies. Hence, on the one hand, financial reporting transparency on EA and GHG emissions is a concept desired by professionals and academics, which can be provided by accounting standard setters. On the other hand, an identified threat for the transparency of environmental disclosures, is the adoption of strategies, even in the presence of mandatory standards, outlined as compliance, dismissal or concealment [27]. Holthausen [28] concludes that the influence of a common set of accounting standards across countries is unlikely to lead to similar financial reporting outcomes. Barbu et al. [29] state that compliance with the IASB standards may differ between entities, and even between countries, due to differences in reporting practices. Thus, there is a suggestion that adopting an explicit accounting treatment does not systematically ensure the transparency of financial reporting. Cowan and Gadenne [30] suggest that companies adopt different approaches to disclose environmental issues when they are potentially subjected to further scrutiny through legal requirements. As a possible concealment strategy, Llena et al. [31] concluded that companies disclose less ‘bad news’, as provisions and contingencies, than ‘good news’, such as investments and expenses. The conclusions of Llena et al. [31] are in line with Deegan and Gordon [32] but disagree with Larrinaga et al. [33] or Adams, et al. [34] that bad news would open companies to challenge. The evidence in the literature that supports that the disclosure of the underlying economics of the EU ETS transactions in the financial reporting—a higher level of transparency—is related to the existence of specific accounting principles is scarce. Based on this significant absence, the following non-directional hypothesis of investigation is formulated:

**H1:**  *Lower levels of transparency of financial reporting on EA and GHG emissions are not associated with the adoption of International Financial Reporting Standards (IFRS)*.

### 2.2. Sample and Data

**Figure 1:**
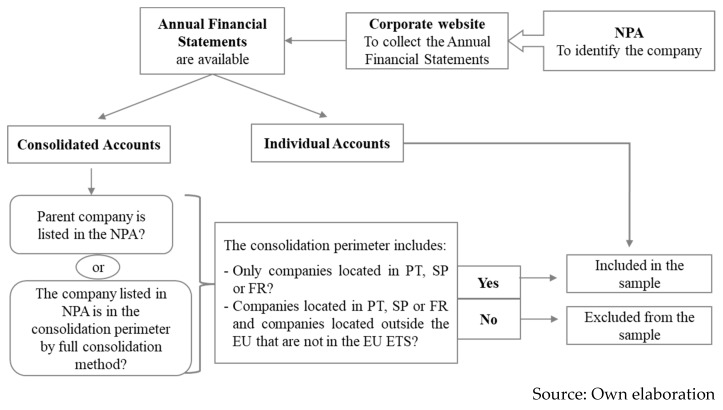
Summary of sample selection. FR, France; PT, Portugal; SP, Spain; NPA, National Plans of Allocation.

The NPAs from Portugal, Spain and France were the starting point for determining the sample as it was the most complete source of installations subjected to the EU ETS. The selection of these countries has been motivated by the purpose of the study which consists of comparing two scenarios of transparency within guidelines based on the (in)existence of specific accounting principles, and taking into consideration the availability of comparable accounting information. Those installations were matched with the respective company-owner. The corporate websites were used to collect the annual financial statements, as they are considered a primary source of information for stakeholders [35]. The selection of the sample mostly depended on the availability of these, in English, Spanish, French or Portuguese. If accessible, the individual annual accounts were collected; in its absence, the consolidated annual accounts were also considered, as long as the company was either the parent company, or it was explicitly mentioned in the Notes as a subsidiary, included by full consolidation method. It was assumed that the parent company rely on the subsidiary to perform under the EU ETS and incorporates the accounting approaches that the subsidiary has on its individual annual accounts—mandatorily or optionally by a gap of the IFRS. The consolidated accounts that included companies that could have installations in other European NPAs were excluded from the sample to prevent the exposure to other domestic regulations that were not identified. Figure 1 presents a diagram for the sample selection.

The period of the study covers 2008 to 2014. Phase I was excluded as it was an experimental period of the EU ETS and also for companies to implement a number of procedures concerning financial reporting, that was much discussed. Previous literature [3,13] struggle to obtain financial data and therefore, it was chosen to commence the study with phase II, following the remaining descriptive studies [11,12,14,16]. It was considered all five years of phase II and the first two years of phase III, which includes the transition to auctioning. The distinction from 2012 to 2013 and 2014 was not made as there has been a postponement in the auctioning of 900 million EA until 2019–2020, due to the surplus of more than 2.1 billion EA in 2013 [20]. In addition, at the beginning of phase III, manufacturing industry received 80% of its EA for free, airlines also receive the large majority of their EA and some power generators, although do not receive any free EA by principle, if the country intends to modernize the sector, then the EU ETS have made some EA available [36].

The final sample is formed by 85 companies, but not for all seven year-period, as it was obtained 548 observations, an unbalanced panel. The representativeness of the sample may be questioned, as the data collected depended on the online availability of annual financial statements. The sample selection is greatly reduced, since a large number of installations that are listed in the NPAs belong to a transnational group of companies that incorporates companies that have installations in other European countries (not exclusively in Portugal, Spain or France), and therefore, apply other financial reporting regulations. This was more significant for French installations, which explains its lack of representativeness. For these companies, only 15 observations are regarding the use of French GAAP, and the results show that they are not disclosing information on EA or GHG emissions, disregarding the domestic regulation. However, this does not invalidate the analysis as it can be assumed that the results are applied to companies with the same characteristics of those in the sample [35].

The content analysis technique was used to evaluate the accounting policies of EA and GHG emissions disclosed in the financial statements [12,13,14,15].

### 2.3. Variables

#### 2.3.1. Dependent Variables

Table 2 (panels A to C) describes the variables that were used as proxies for transparency of financial reporting on EA and GHG emissions. Panel A presents the eight accounting policies that were collected from the Notes to the annual accounts. These categorical variables are the basis for the formulation of the transparency variables: Accounting approaches and indexes of the disclosure, summarized in panels B and C, respectively.

The variables defined in panel A of Table 2 are a summary of the questions raised in the literature review for the recognition and measurement of EA and GHG emissions [11,12,13,14,38] supported with the issues prescribed by French, Spanish and Portuguese standard setters. Eight categorical variables were determined: I1. Initial recognition of free EA, I2. Counterpart on the initial recognition of free EA, I3. Initial measurement of free EA, I4. Subsequent measurement of EA, I5. Recognition of GHG emissions, I6. Measurement of GHG emissions, I7. Recognition of GHG emissions over-allocated EA, I8. Recognition of EA acquired to cover GHG emissions over cap. These categorical variables enable to identify the adopted accounting approach in each company-year observation, by the joint answers given to the accounting policies—I1 to I7 (panel B of Table 2). Each accounting approach is a dichotomous variable that takes the value 1 if it was identified and 0 otherwise. The following accounting approaches were identified: the IFRIC 3 cost model, the government grant approach as described by Ernst and Young [23] (an approach that is similar to the ‘cost with balance at market value’ mentioned by Lovell et al. [11], to ‘modified IFRIC 3 approach’ mentioned by Black [14], and also to ICAC Resolution of 8 of February as mentioned in Section 2.1.1—Table 1); the net liability approach; the French inventory approach defined by ANC in 2012; the Spanish approach (until 2016) defined by ICAC in Resolution 8 of February of 2006; the Portuguese approach defined by CNC in the appendix to AFRS 26; and the approach of disclosing off-balance sheet policies, and non-disclosure. Panel C of Table 2 shows self-elaborated indexes as proxies for the extent of disclosures that reflect aspects related to the EU ETS compliance transactions, as frequently used in the literature [12,37,39]. The indexes were based in the eight categorical variables, that were transformed into dichotomous variables (I1.dummy to I8.dummy) that take the value 1 if some categorical information was disclosed and 0 for the cases of non-disclosure and disclosure of off-balance sheet policies (coded as 0 and 99, respectively, in tables of Appendix A). The index of disclosure (ID) is the sum of the eight dummy variables (I1.dummy to I8.dummy) and takes values from 0 to 8. The level of transparency of EA and GHG emissions is also assessed by ‘good news’ and ‘bad news’: The ID related to EA (I1 to I4) is seen as a ‘good news’ once it gives the company free allowances and accordingly to market rules; the ID related to GHG emissions (I5 to I8) is seen as ‘bad news’ as it internalizes ‘the cost of polluting’.

#### 2.3.2. Independent Variables: Treatment and Control

The explanatory variable is the basis of presentation of financial reporting (BoPFS). The definition of the sample exposes companies to the preparation of financial reporting on accordance with IFRS, either mandatory or optional, or with national accounting standards. Previous literature [13,35,40,41,42] state that accounting practices are distinguished by the standard set that includes specific regulation on the subject versus those leading to professional judgment. Thus, the variable basis of presentation of financial statements under IFRS (BoPFS.IFRS) is a dummy variable that takes the value 1 for observations in IFRS, and the value 0 to represent observations in national standards (Portuguese, Spanish or French). Some caution has to be considered because the consolidated annual accounts of the parent company may be prepared within IFRS, although national regulation obligates the subsidiary to prepare individual annual accounts within national standards (this is the case of Spanish and French regulation, but not Portuguese). Considering that the IFRS have a specific regulatory gap, but the Conceptual Frameworks of Portuguese, Spanish and French standard setters do not contradict the IASB’s, there is an argument for the potential influence of the subsidiary financial reporting on the consolidated. Allini et al. [9] present a similar argument to assess whether domestic standard setters have influence in the consolidated accounts. It is therefore important to consider that the majority of observations are based in IFRS reporting and that the use of the French standards is residual (Table 3).

Regarding control variables, we expect that size, profitability, financial risk, external supervision and industry provide good factors to control the transparency of financial reporting on EA and GHG emissions. Size is considered as a positive influence on the disclosure of information related to GHG emissions [39,43,44,45], since it emphasizes that large companies are politically more sensitive, facing pressures that smaller ones do not have. There are several measures to proxy size, and we choose the logarithm of total assets as Giner [40] and Gallego-Álvarez et al. [35]. Companies that are more profitable may be more transparent about their activities, and therefore, are expected to present more disclosures to justify good performance. Several studies simultaneously use both return on assets (ROA) and return on equity (ROE) to control for profitability (most observations are from unlisted companies, and therefore, a proxy for profitability could not be market-based), as ROA reflects an operational performance and ROE a financial performance [40,44,46]. We also introduced return on sales (ROS) as it represents the short-term return [47]. Leverage ratios are used to inform about default risks that may lead to avoiding investment and financing. They are often used as a covenant, which leads managers to adopt accounting policies that avoid an inconvenient outcome [44]. There are also several measures used in the literature, as we choose to include debt-to-equity ratio [35,44] and debt-to-assets ratio [24,45]. These control variables (size, ROA, ROE, ROS; debt-to-equity and debt-to-assets) were categorized into ‘1—small’, ‘2—medium’ or ‘3—large’. Class 1 is up to 25th percentile, class 2 is between the 25th percentile (inclusive) and the 75th percentile (inclusive), and from the 75th percentile, it was categorized as class 3.

The presentation of an independent auditor report certifies the reliability and relevance of accounting practice, thus providing more credibility to financial statements [48]. It is expected that the presentation of an independent auditor report has a positive influence on transparency. The variable Audit is a dichotomous variable that takes the value 1 if the annual report incorporates the auditor’s report and the value 0 if not. We realize that unlike the remaining control variables, the presence of auditors’ report as a dummy does not capture changes between years.

The literature review provided arguments that practices regarding financial reporting may vary across the industry. On another perspective, it has to be considered that the legal requirements for the financial reporting regarding EA and GHG emissions are similar for all companies in the sample. An initial analysis considered industry as a control variable, coded as a dummy for each industry. Nevertheless, the multivariate analysis showed multicollinearity issues, which led us to omit it from the model. Table 4 presents the frequencies for these variables.

#### 2.3.3. Model and Econometric Analysis

The proposed econometric model intends to assess if the exposure to IFRS harms the transparency of financial reporting on EA and GHG emissions:

**Model 1:** LevelTransparency_i*t*_ = ∫(Basis of presentation of financial reporting, control variables)_i*t*_

Equations (1) and (2) were designed to verify the influence of the IFRS in the transparency of financial reporting.

(1) IDit= β0 + β1 BoPFS.IFRSit +øXit+ εit,

(2) AAit= β0 + β1 BoPFS.IFRSit +øXit+ εit,

where i and t are, respectively, company and year; ID represents the indexes of the disclosure: ID, ID.EA, ID.eGHG; AA embodies the identified accounting approach; BoPFS.IFRS is the adoption of the IFRS as BoPFS; X corresponds to control variables: Size, Profitability, Leverage and Audit; ε is the error term.

In order to assess the transparency of the accounting policies disclosed by BoPFS, a univariate analysis was carried out, mainly focused on measures of frequency and summary statistics.

For Equation (1), the developments in panel data techniques allow to independently treat the dynamics of the individuals in a period of time and eliminates the bias of the aggregation, which is known as individual effects or unobservable heterogeneity [49,50]. The consistency of parameter estimators, and consequently, the validity of the economic interpretation, rely on the correct functional form specification and controlling for unobserved heterogeneity [51]. The Hausman test compares the use of two estimators (fixed or random effects model), which depends on whether there are (or not) individual effects. However, Wald’s’ modified test indicated heteroscedasticity, and therefore, standard error estimates had to be corrected for the homoscedasticity condition. It was used a Panel Corrected Standard Errors (PCSE) regression. The use of this estimator also allowed to correct issues of first order autocorrelation, whenever detected, by Wooldridge test. When it was verified heteroscedasticity and first-order autocorrelation, the correction was made through Prais–Winsten estimator. Moundigbaye et al. [52] state that PCSE preserves the (Prais–Winsten) weighting of observations for autocorrelation, but uses a sandwich estimator to incorporate cross-sectional dependence when calculating standard errors.

Equation 2 is based on a model of binary choice. It expresses the likelihood of the company adopting an accounting approach, depending on the regulatory BoPFS. This is a logistic regression model in which the normal distribution is replaced by the logistic [53]. However, the identification of certain accounting approaches (IFRIC 3 cost model, net liability approach) ischaracterized a rare event (see panel A of Appendix B). In order to minimize the bias of the maximum likelihood estimation against the reduced sample, Firth’s [54] Penalized Maximum Likelihood Estimation was used, because in cases of ‘separation’, it allows convergence for finite estimates.

The next chapter presents the results and discussion.

## 3. Results and Discussion

### 3.1. Univariate Analysis

The characterization of the sample by country and by year suggests that throughout the years, there is consistency on the disclosure of the accounting policies, as it is supposed. It should be noted that both Portuguese and French companies may present less consistency, due to alterations in accounting regulation. More specifically, the majority of Spanish companies disclose the recognition of the EA, while French companies are those that present a large frequency of lack of disclosures of such information (for both disclosures on EA and GHG emissions). Portuguese companies are present more discretion, which is less evident from the year of 2010 on regarding GHG emissions, when the recognition of it as the amortization of the EA, using FiFo, becomes part of accounting regulation.

Appendix A presents the frequencies of each accounting policy for the recognition and measurement of EA and GHG emissions. It is assessed that within the application of the Spanish standards, 98,75% disclose the recognition of EA, but in the IFRS adoption, only 68,82% do it. Whilst, 18,26% of the IFRS observations do not disclose information about EA and 9,83% disclose the adoption of off-balance sheet policies. The analysis shows the following tendencies for EA: Recognition as an intangible asset with a counterpart in owners’ equity or deferred income, at market value; subsequently, EA are preferably measured at cost fewer impairment losses. PWC and IETA [3], Steenkamp et al. [12] and Black [14] concluded that most companies initially measure the EA at nil value, which is not validated in this study. One possible explanation for recognition in owner’s equity and initial measurement at market value is the influence of Portuguese and Spanish standards. Regarding GHG emissions, in line with what was verified for EA, almost 20% of the observations in IFRS discloses the adoption of off-balance sheet policies. The results for the basis of measurement of the GHG emissions identify 13 bases. Similarly, Warwick and Ng [13] found 11 bases of measurement for GHG emissions. Nevertheless, the most common bases are the cost and the carrying amount of the EA, as PWC and IETA [3] and Ayaz [15] also conclude. It may be related to the high frequencies of initial measurement for EA at market value. It should be noted that there is more diversity within the use of the IFRS and that, for all accounting policies, the observations based on French standards only report non-disclosure.

Appendix B (panels A and B) presents the mean and standard deviation for the accounting approaches and indexes of the disclosure. The most used accounting approach under the IFRS is government grant—as identified by Ernst and Young [23], followed by not disclosing information on EA or GHG emissions. Under the adoption of the IFRS and Portuguese standards, the report of off-balance sheet policies suggests the use of strategies of concealment. The index of disclosure presents a mean of approximately five items, increased when it is based in national standards, particularly Spanish, and decreased when the bases are the IFRS. The standard deviation regarding the IFRS indexes provides evidence of the disparity in the levels of transparency. These results are in line with Larrinaga [21] that argued the lack of guidance from the IASB as a motivating factor for the adoption of discretionary views. The companies that present their annual accounts using the Spanish standard are the most transparent, as well as those with less discrepancy in accounting policies for EA and GHG emissions. The results also suggest that whether companies apply IFRS or national standards, the tendency is to disclose more information on EA than on GHG emissions. However, this does not mean dismissal of the standard or a strategy of concealment. The index of ‘bad news’ includes the recognition of GHG emissions above EA held and the acquisition of EA, which may not be a necessity of disclosure, although it would be more transparent to present an accounting policy for it. The difference is more significant for the Portuguese standard, which we attribute to the prescribed accounting treatment for GHG emissions–—the amortization of the EA and not the recognition of a liability.

As expected, when specific accounting treatment is missing—in an IFRS context—more approaches are allowed, thus improving the level of transparency of financial reporting of EA and GHG emissions. However, even transparency under specific accounting regulations is not consistent (in an analysis across time). The changes on national regulations in the period 2008-2014 may have contributed to this situation (as companies that apply Spanish GAAP—a steady regulation in such period, present a lesser number of adopted accounting approaches, and even so, government grant and ICAC approaches are similar). Discretion is, therefore, a situation that the existence of regulation may minimize, but to stabilize the levels of transparency of financial reporting, thus reducing discretion, we believe to be necessary a common approach. Without that, the companies that are under unspecific accounting treatments will have the possibility to present the transactions with different levels of transparency (even across time); and those with specific accounting treatments, will also benefit with the doubt created by the international community. Given this situation, the IASB should create a more stable situation, that we hope it comes to support a full recognition of the assets and liabilities, hence ensuring more transparency and less discretion in the financial reports.

### 3.2. Multivariate Analysis

Appendix D presents the results for Equation (1). It should be noted that industry variables presented a variance inflation factor (VIF) above 10 (excluding aviation). As dummy variables, it is understandable that they present correlation among them (see Appendix C). These results do not affect the coefficient of the explanatory variable, as it was decided to remove them from the models. For models 1 and 3 it was used a linear regression heteroscedastic PCSE and for model 2, to deal with simultaneous heteroscedastic and first order autocorrelation, it was used a Prais–Winsten regression, heteroscedastic PCSE. The following post-estimation tests are presented: Wald tests to verify the significance of time dummy variables—identified as i.Year; the Wald modified test to assess the presence of heteroscedasticity—identified as Waldchi2; and the Wooldridge test verifies the presence of first-order autocorrelation—identified as AR(1).

The results show that the application of the IFRS has a negative effect on the indexes of the disclosure. That effect is stronger regarding disclosures of EA than GHG emissions, suggesting that there is a tendency to incorporate less information about the EA in IFRS reporting than information on GHG emissions. Control variables, such as ROS, debt-to-equity, or auditors’ report have a positive influence on the indexes of the disclosure.

Appendix E shows the results for Equation (2) using the Penalized Maximum Likelihood Estimation of Firth’s [54]. Models 1 and 4 on the probability of disclosure the IFRIC 3—cost model and inventories approach are not statistically significant. Model 5 regarding the probability of disclosing the ICAC model (from 2006), although statistically significant, presents no significant probability of being influenced by the use of the IFRS. Somewhat similarly, Moneva and Llena [55] provide empirical evidence that, in the Spanish context, there are no significant differences regarding environmental disclosures between large quoted and non-quoted companies. The probability of reporting the government grant and the net liability approaches is positively and strongly associated with the adoption of the IFRS. The same positive direction applies to the disclosure of off-balance sheet policies and also, for non-disclosure. The probability of adopting the AFRS 26 approach is negatively associated with the IFRS, suggesting that only under the adoption of Portuguese standards this approach has been disclosed. Monteiro and Vilas Boas [39] that concluded that although AFRS 26 is legally imposed for unlisted companies, listed companies also presented a higher index of disclosures on EA. Therefore, it may be implied that listed companies disclose information on EA and GHG emissions although not under the direct influence of the Portuguese standard, are concerned in presenting financial information on the participation on the EU ETS. The probability of reporting the government grant and the net liability approaches is positively and strongly associated with the adoption of the IFRS. The same positive direction applies to the disclosure of off-balance sheet policies, and also, for non-disclosure

The results obtained suggest that the transparency of financial reporting on EA and GHG emissions are negatively influence by the IFRS. Hence, H1 is rejected because there is an association regarding the indexes of the disclosure and concerning four specific approaches: Non-disclosure, off-balance sheet recognition, government grant approach and net liability approach. This is due to the fact that the IASB does not specifically state its position on the EU ETS transactions, but the IFRSs provide several answers to include EU ETS transactions in the financial statements that do not include its omission (within IAS 38, IAS 20, IAS 37 or IFRS 9). The levels of transparency of financial reporting on EA and GHG emissions are lower in companies that adopt the IFRS. The use of the IFRS is positively related to the probability to use accounting approaches that minimize transparency, such as net liability approach, off-balance sheet policies or non-disclosure. However, it is also positively associated with the government grant approach, a gross approach that provides more information in financial reporting. Hence, there is an association between transparency and accounting regulation, but there is no evidence of a significant reduction of the levels of transparency of financial reporting prepared under the IFRS. The existence of mandatory regulation seems to be sufficient to provide a relative increase in the transparency of financial reporting. This is in contrast with Giner [40] conclusion that legislation appears to produce a strong increase in disclosure, even before being compulsory. Barbu et al. [29] stated that compliance (with IASB standards) might differ between companies and even between countries, due to differences in reporting practices. So, even in environments where the standard setter did not issue specific regulation, the transparency of financial reporting on EA and GHG emissions may be positively influenced by national regulation, but not sufficient to disregard other influencing factors. The suggestion that companies could use strategies, such as disclose more ‘good news’, related to EA, than with ‘bad news’, regarding GHG emissions [31,33], was not verified. Therefore, the lack of specific legislation does not influence companies to disclose more accounting policies related to EA than with GHG emissions. Therefore, companies are disclosing accounting practices on both “the asset” as “the liability”, possibly to compensate the ‘cost of polluting’ with the recognition of licenses that permit to emit a given number of GHG for free. Generally, it can be argued that lower levels of transparency regarding EA and GHG emissions are associated with this lack of specific regulation, but is not as significant as it could be expected.

In summary, we believe that as regulation may not be decisive in the disclosure process, it could increase the levels of transparency and particularly the possibility of comparison amongst companies. The results show that the many approaches taken by IFRS companies include mainly approaches of non-disclosure, off-balance sheet recognition and a net liability approach (although some choose government grant disclosure). A clear position by the IASB would help to reduce that discretion and to increase the transparency of the financial reporting that represents EU ETS transactions. The next chapter presents the conclusions and contributions of the study, as well as its limitations.

## 4. Conclusions

### 4.1. Conclusions and Contribution

This study aims to provide evidence of the influence of accounting regulation in the transparency of financial reporting on EA and GHG emissions. It was considered the perspectives from the IASB, national standard setters and the reporting practices commonly adopted by companies, accordingly to previous literature. This issue arises from the lack of specific guidance in the IFRS, which motivated European national standard setters to overcome it, and led professionals to exercise their professional judgment. The Portuguese and Spanish standard setters adopted a gross approach, similar to the IFRIC 3—although this is not a condition to adopt a gross approach; the French standard setter changed the fundamentals of the prescribed approach in 2012 (for periods beginning at 1/1/2013 or after), from a gross to a net approach. We trust that gross approaches provide better levels of transparency and provide stakeholders with relevant and faithfully represented information on the companies’ exposure to the EU ETS. Notwithstanding, as a high-quality standard set, the IFRS provide a basis to adopt policies that meet those characteristics.

Multiple accounting approaches are identified, mainly within the adoption of IFRS. The transparency of financial reporting based in IFRS is positively influenced by the probability of adopting approaches such as the government grant (gross approach) and net liability approach; and negatively influenced by the probability of disclosing off-balance sheet and practices of non-disclosure. Hence, the companies that adopt the IFRS tend to disclose less information than companies that apply regulations with specific accounting principles. Moreover, it is within the IFRS that it is verified more dispersion in the reported information, implying that there is not a commonly accepted framework for disclosures. As a conclusion, this study provides evidence that the existence of specific mandatory regulation is sufficient to provide a relative increase on the transparency of financial reporting, but other factors have to be considered for a more comprehensive analysis. Companies reporting on the basis of the IFRS have looked forms of reporting to their stakeholders the exposure to the EU ETS; however, we do not see it as sufficient. The lack of specific regulation prejudices transparency (allowing the not disclosing or the disclosure of off-balance sheet policies) but also leads to total discretion on the accounting approaches that are adopted. The results suggest that such discretion is a situation that the existence of consistent regulation may minimize, but to stabilize the levels of transparency of financial reporting between companies, thus reducing discretion, we believe to be necessary a common approach. Without that, the companies that are under unspecific accounting treatments will have the possibility to present the transactions with different levels of transparency; and those with specific accounting treatments will also benefit with the doubt created by the surrounding community. This is a fundamental reason that urges the regulation of IFRS transactions, in line with the international harmonization process. The IASB is, ultimately, responsible for creating a more stable situation, that we hope it supports a full recognition of the assets and liabilities, hence ensuring more transparency and less discretion in the financial reports.

Given the reduced number of studies concerning such matters, this research highlighted that transparency in financial reporting should be focused in the underlying economics of the EU ETS transactions: The full recognition of the related assets and liabilities. The lack of comparability between companies, the strategies adopted for disclosing some information (such as off-balance sheet policies) and the lack of disclosure, harm the desired transparency for financial reporting.

### 4.2. Limitations

A note of caution is needed because of the inherent limitations. The first is related to the availability of annual financial statements. This led us not to consider important determinants as listed/unlisted companies as some of the annual reports were only available for consolidated reporting. In such cases, the individual company, that was the receiver of the EA was not listed, although the parent company (and the reporting entity) was, . Hence, the consolidated financial disclosures were considered as the accounting treatment adopted by the individual company, but the inherent characteristics (as size or profitability) were regarding the individual company. Second, the use of content analysis may result in bias, as sometimes it is necessary to make some subjective judgements. This is regarding the analysis of the perimeter of consolidation, to include a wider scope of companies, and to assess the proxies of transparency. Finally, we acknowledge that the use of all companies listed in the NPAs with available annual financial statements may potentially include information within materiality issues that increments the adoption of off-balance sheet policies and non-disclosure.

## Figures and Tables

**Table 1:** Summary of Accounting Approaches for Emission Allowances (EA) and Greenhouse Gas (GHG) Emissions.

	IFRIC 3	CNC—Portugal ^1^		ICAC—Spain ^2^		ANC—France ^3^	
		Before 1 January 2010	After 1 January 2010	Before 1 January 2016	After 1 January 2016	Before 1 January 2013	After 1 January 2013
**Recognition of EA**	Intangible assets/Government Grant (Deferred income)	Intangible assets/Government Grant (Deferred income)	Intangible assets/Government Grant (Owners’ Equity)	Intangible assets/Government Grant (Owners’ Equity)	Inventory ^4^/Government Grant (Owners’ Equity)	Intangible assets/Liability—*Quotas d’émission alloués par l’État*	Inventory
**Initial measurement of EA**	Market value	Fair value	Fair value	Market value	Market value (fair value)	Market value	Nil value
**Subsequent measurement of EA**	Cost model or Revaluation model (without amortizations)	Cost less accumulated impairments (extraordinary amortization)	Cost less accumulated amortizations	Cost less accumulated impairments	Cost less accumulated impairments	Cost or Acquisition cost of equivalents (best estimate)	--
**Recognition and measurement of GHG emissions**	Operational Loss/Provisions ^1^ using best estimate (usually the market price)	Operational Loss/Provisions using *First in First out* (FIFO)	Amortization loss/Accumulated Amortizations using *First in First out* (FIFO)	Operational Loss/Provisions using: 1) the carrying amount of the EA held (proportion of GHG emissions of the period to the total GHG emissions) 2) weighted average cost (WAC) of the remaining EA.		Loss/Liability using the initial value of EA	Operational loss/Inventory using FIFO or WAC
	Recognize the government grant (as income) over the periods in which the related expenses are intended to offset						
**GHG emissions above detained EA**	Loss/Provision using best estimate (when the estimate of GHG emissions exceeds the carrying amount of the EA held for purposes of compliance) (In the case of Portugal, before 1 January 2010, was prescribed the fair value for this measurement)					Loss/Liability using the close value of EA	Liability at acquisition cost for EA necessary to cover the GHG emissions
^1^ The Portuguese legislation **(Comissão de Normalização Contabilística-CNC)** is presented with two accounting treatments, due to the legislative amendment introduced for periods beginning on or after 1 January, 2010. The accounting treatment in force until 1 January, 2010, was introduced by Technical Interpretation (TI) 4. For periods after 1 January, 2010, Notice 15654/2009 of 7 September (modified by Notice 8256/2015 of July 29) changes the accounting treatment provided for in that TI. ^2^ The Spanish legislation (Instituto de Contabilidad y Auditoría de Cuentas-ICAC) is presented with two accounting treatments, due to the legislative amendment introduced for periods beginning on or after 1 January, 2016. The accounting treatment in force until January 1, 2016, was introduced by the Resolution of 8 February, 2006, and by the Resolution of 23 May, 2013. Royal Decree 602/2016 changes the accounting treatment of the aforementioned Resolutions, for periods starting from 1 January 2016, but only as regards the nature of the EA held to comply with the obligation to settle the GHG emissions incurred. ^3^ The French legislation (Autorité des Normes Comptables-ANC)is presented with two accounting treatments, due to the legislative amendment introduced for periods beginning on or after 1 January, 2013. The *Avis nº 2004-C du 23 mars du Comité de urgence* established the accounting treatment recommended until 1 January, 2013. *Réglement no. 2012-03 of 4 Octobre 2012* changes the accounting treatment of GHG emission allowances and assimilated units, but the comparison refers only to the production model and excludes the trading model. ^4^ EA to be consumed after a period of one year shall be presented in a separated line.							

**Source:** Own elaboratio.

**Table 2:** Variable definitions.

**Panel A—Accounting policies for recognition and measurement of EA and GHG emissions**							
**Categorical variables**						**Abbreviation**	
I1. Initial recognition of EA						Rec EA	
I2. Counterpart on the initial recognition of EA						Count EA	
I3. Initial measurement of EA						Initial Meas EA	
I4. Subsequent measurement of EA						Subseq Meas EA	
I5. Recognition of GHG emissions						Rec GHG emissions	
I6. Measurement of GHG emissions						Meas GHG emissions	
I7. Recognition of GHG emissions over-allocated EA						Rec GHGe over EA	
I8. Recognition of EA acquired to cover GHG emissions over cap						Rec EA acq	
**Panel B—Identification of Accounting Approaches**							
**Approach**	I1	I2	I3	I4	I5	I6	I7
**IFRIC3_cost**	1	3-5-6-7	3-4-5	3	3-6	4-7	
**GovGrant**	1	3-5-6-7	3-4-5	3	3-6	9-11-12-13	
**NetLiab**			1-6-7-99		7-0		1-2-3-4
**Inventory**	2		1-6-7	1-6	2-3	^a^ 1-2-5-6-9-10-11-12-13	
**ICAC**	1	1-7	3-4-5	3	3-6	4-5-6-10	
**AFRS26 ^b^**	1	1-7	2-3-4-5	2-4		2	
**NoRec**	99		99		7		5
**NoDisc**	0		0		0		0
The accounting approach is a dichotomous variable, that takes the value 1 if it is identified by the joint answers of each categorical variable (I1 to I7)—representing accounting policies, and 0 otherwise. Each categorical variable may present one or more possibilities to be considered that a certain accounting approach is being adopted. The numbers showed below each categorical variable correspond to the category expressed on Appendix A. The abbreviations for the identified accounting approaches are as follows: IFRIC 3—cost model (IFRIC3_cost), government grant approach (GovGrant) as exposed by Ernst and Young [37], net liability approach (NetLiab), the French inventory approach (Inventory) as exposed by ANC in 2012, the Spanish approach exposed in Resolution 8 of February of 2006 (ICAC), the Portuguese approach exposed in the appendix to Accounting and Financial Reporting Standard (AFRS) 26 (hereafter AFRS26), the approach of disclosing off-balance sheet policies (NoRec), and the approach of non-disclosure of EA or GHG emissions (NoDisc). A I6 for inventory approach presents a variety of options in content analysis (1-2-5-6-9-10-11-12-13 in Appendix A), but there are only six observations that fulfil the previous criteria. It discloses I6 as the carrying amount of EA held. B I7 was not considered to identify the approach because the disclosure may not have happened if GHG emissions are below EA allocated for free and held for compliance.							
**Panel C—Indexes of the Disclosure**							
**Variable**		**Type**		**Description**		**Measurement**	
*ID*		Levels of transparency		Index of disclosure		Takes values from 0 to 8 (is the sum of I1.dummy to I8.dummy)	
*ID.IFRS*				Index of disclosure in an IFRS basis			
*ID.NatGAAP*				Index of disclosure in a national GAAP basis			
*ID.EA*		Levels of transparency—Good *vs* Bad news		Index of disclosure of *good news*		Takes values from 0 to 4 (is the sum of I1.dummy to I4.dummy for EA; is the sum of I5.dummy to I8.dummy for GHG emissions)	
*ID.IFRS.EA*				Index of disclosure of *good news* in IFRS			
*ID.NatGAAP.EA*				Index of disclosure of *good news* in national GAAP			
*ID.eGHG*				Index of disclosure of *bad news*			
*ID.IFRS.eGHG*				Index of disclosure of *bad news* in IFRS			
*ID.NatGAAP.eGHG*				Index of disclosure of *bad news* in national GAAP			

Source: Own elaboration.

**Table 3:** Frequencies on the basis of presentation of financial reporting (BoPFS) per country.

BoPFS	Spain		France		Portugal		Total	
	Freq	Perc	Freq	Perc	Freq	Perc	Freq	Perc
IFRS	215	72,88	73	82,95	68	41,21	356	64.96
Spanish GAAP	80	27,12					80	14.60
French GAAP			15	17,05			15	2,74
Portuguese GAAP					97	58,79	97	17.70
**Total**	**295**	**100,00**	**88**	**100,00**	**165**	**100,00**	**548**	**100.00**

**Table 4:** Frequencies for Industry.

Industry	Freq.	Percent
**Aviation**	9	1.64
**Cement**	56	10.22
**Combustion**	231	42.15
**Pulp and paper**	102	18.61
**Production of electricity**	67	12.23
**Oil refineries**	6	1.09
**Steel**	42	7.66
**Glass**	35	6.39
	**548**	**100.00**

## Appendix A—Frequencies of the Accounting Policies Regarding EA and GHG Emissions

See Table 2 for variable descriptions.

**Table:**

	**IFRS**		**Spanish GAAP**		**French GAAP**		**Portuguese GAAP**	
	**Freq**	**Perc**	**Freq**	**Perc**	**Freq**	**Perc**	**Freq**	**Perc**
**Are EAs’ recognized?**								
Yes	245	68.82	79	98.75			85	87.63
No	35	9.83					2	2.06
Only when GHG emissions exceed EA held	9	2.53						
Do not have EA	2	0.56					1	1.03
Not disclosed	65	18.26	1	1.25	15	100.00	9	9.28
**I1. Rec EA**								
1—Intangible Asset	222	62.36	79	98.75			82	84.54
2—Inventory	22	6.18						
3—Financial Asset							3	3.09
4—Other current asset	5	1.40						
0—Not disclosed	70	19.66	1	1.25	15	100.00	9	9.28
99- Not applied	37	10.39					3	3.09
**I2. Count EA**								
1—Owners’ Equity			65	81.25			64	65.98
2—Other non-current liability	7	1.97						
3—Liability							4	4.12
4—Financial liability							3	3.09
5—Liability—EA allocated	4	1.12						
6—Deferred income	145	40.73					9	9.28
7—Government grant	45	12.64	7	8.75				
8—Grant reduced to asset carrying amount	11	3.09						
9—Income—Government grant	5	1.40						
10—Operational income	7	1.97						
0—Not disclosed	95	26.69	8	10.00	15	100.00	14	14.43
99—Not applied	37	10.39					3	3.09
**I3. Initial Meas EA**								
1—Acquisition cost	6	1.69	2	2.50				
2—Fair value							15	15.46
3—Market value	175	49.16	64	80.00			64	65.98
4—Price	7	1.97						
5—Quoted average price	7	1.97						
6—Zero	41	11.52						
7—Symbolic value	6	1.69	7	8.75				
0—Not disclosed	77	21.63	7	8.75	15	100.00	15	15.46
99—Not applied	37	10.39					3	3.09
**I4. Subseq Meas EA**								
1—Historical cost	58	16.29	4	5.00			6	6.19
2—Cost less accumulated amortisations							15	15.46
3—Cost less accumulated impairment losses	120	33.71	43	53.75			5	5.15
4—Cost model							25	25.77
5—*First in First Out*	2	0.56						
6—Cost or net realisable value, the lower	6	1.69						
7—Fair value	12	3.37					20	20.62
8—Revaluation model							5	5.15
9—*Net liability approach*			7	8.75			1	1.03
10—*Net asset approach*	7	1.97						
0—Not disclosed	114	32.02	26	32.50	15	100,00	17	17.53
99—Not applied	37	10.39					3	3.09
**I5. Rec GHG emissions**								
1—Amortization expenses							39	40.21
2—Cost of consumed materials	2	0.56						
3—Other operational losses	14	3.93					6	6.19
4—Liability	11	3.09					4	4.12
5—Financial Liability							3	3.09
6—Provision	182	51.12	79	98.75			14	14.43
7—No recognition	70	19.66					18	18.56
0—Not disclosed	77	21.63	1	1.25	15	100,00	13	13.40
**I6. Mens GHG emissions**								
1—Cost			26	32.50				
2—*First in First Out*	2	0.56					32	32.99
3—Initial price	7	1.97						
4—Average price of EA	7	1.97					3	3.09
5—Average cost of EA + Acq. Cost EA	6	1.69						
6—Average cost of EA + Best estimate to acquire	7	1.97	7	8.75				
7—Fuel consumption + average price of EA			3	3.75				
8—Cancelation of the obligation order			7	8.75				
9—Carrying amount (CA) EA	68	19.10	11	13.75			1	1.03
10—CA EA + average price of EA + best estimate	14	3.93	12	15.00				
11—CA EA + Best estimate to acquire	40	11.24						
12—CA EA + price at closing	17	4.78						
13—CA EA + market value	4	1.12						
0—Not disclosed	117	32.87	14	17.50	15	100.00	43	44.33
99—Not applied	67	18.82					18	18.56
**I7. Rec GHGe over EA**								
1—Expense accrual							2	2.06
2—Liability	19	5.34						
3—Financial Liability	14	3.93					14	14.43
4—Provision	109	30.62	28	35.00			44	45.36
5—No recognition	28	7.87					11	11.34
0—Not disclosed	186	52.25	52	65.00	15	100.00	26	26.80
**I8. Rec EA acq**								
1—Intangible Asset	185	51.97	67	83.75			45	46.39
2—Inventory	27	7.58						
3—Never acquired EA							14	14.43
0—Not disclosed	144	40.45	13	16.25	15	100.00	38	39.18

## Appendix B—Levels of Transparency of EA and GHG Emissions Financial Reporting

See Table 2 for variable definitions. Std. Dev. is the standard deviation.

**Panel A—Descriptive summary of accounting approaches by BoPFS.**

**Table:**

**Accounting Approach**	**Full sample** **(Obs: 548)**		**IFRS** **(obs = 356)**		**Spanish** **(obs = 80)**		**French** **(obs = 15)**		**Portuguese** **(obs = 97)**	
	**Mean**	**Std. Dev.**	**Mean**	**Std. Dev.**	**Mean**	**Std. Dev.**	**Mean**	**Std. Dev.**	**Mean**	**Std. Dev.**
IFRIC3_cost	0,0128	0,1124	0,0197	0,1390	0,0000	0,0000	0,0000	0,0000	0,0000	0,0000
GovGrant	0,1569	0,3641	0,2809	0,4501	0,0625	0,2436	0,0000	0,0000	0,0000	0,0000
Inventory	0,0511	0,2204	0,0787	0,2696	0,0000	0,0000	0,0000	0,0000	0,0000	0,0000
*NetLiab*	0,0109	0,1042	0,0169	0,1289	0,0000	0,0000	0,0000	0,0000	0,0000	0,0000
ICAC	0,0438	0,2048	0,0393	0,1946	0,0875	0,2843	0,0000	0,0000	0,0309	0,1740
AFRS26	0,0511	0,2204	0,0000	0,0000	0,0000	0,0000	0,0000	0,0000	0,2887	0,4555
NoRec	0,0420	0,2007	0,0590	0,2359	0,0000	0,0000	0,0000	0,0000	0,0206	0,1428
NoDisc	0,1369	0,3440	0,1433	0,3508	0,0125	0,1118	1,0000	0,0000	0,0825	0,2765

**Panel B—Descriptive summary of indexes of disclosure by BoPFS**

**Table:**

**Variable**	**Full sample** **(Obs: 548)**		**IFRS GAAP** **(Obs: 356)**		**Spanish GAAP** **(Obs: 80)**		**Portuguese GAAP** **(obs = 97)**		**Min**	**Max**
	**Mean**	**Std. Dev.**	**Mean**	**Std. Dev.**	**Mean**	**Std. Dev.**	**Mean**	**Std. Dev.**		
***I1.dummy***	**0.7442**	0.4367	0.6994	0.4591	0.9875	0.1118	0.8763	0.3310	0	1
*I2.dummy*	0.6762	0.4683	0.6292	0.4837	0.9000	0.3019	0.8247	0.3822	0	1
*I3.dummy*	0.7102	0.4541	0.6798	0.4672	0.9125	0.2843	0.8144	0.3908	0	1
*I4.dummy*	0.6064	0.4890	0.5758	0.4949	0.6750	0.4713	0.7938	0.4067	0	1
*I5.dummy*	0.6386	0.4808	0.5871	0.4931	0.9875	0.1118	0.6804	0.4687	0	1
*I6.dummy*	0.4955	0.5004	0.4831	0.5004	0.8250	0.3824	0.3711	0.4856	0	1
*I7.dummy*	0.4132	0.4929	0.3989	0.4904	0.3500	0.4800	0.6186	0.4883	0	1
*I8.dummy*	0.6100	0.4882	0.5955	0.4915	0.8375	0.3712	0.6082	0.4907	0	1
ID	4.9543	2.9162	4.6489	3.0743	6.4750	1.3685	5.5876	2.3352	0	8
ID.EA	2.7719	1.6418	2.5843	1.7235	3.4750	0.8711	3.3093	1.3099	0	4
ID.eGHG	2.1823	1.4773	2.0646	1.5305	3.0000	0.9808	2.2784	1.2726	0	4

## Appendix C—Correlation Matrix

See Table 2 for variable definitions.

**Panel A—Spearman’s correlation matrix (Equation (1)).**

**Table:**

	**(1)**	**(2)**	**(3)**	**(4)**	**(5)**	**(6)**	**(7)**	**(8)**	**(9)**	**(10)**	**(11)**	**(12)**	**(13)**	**(14)**	**(15)**	**(16)**	**(17)**	**(18)**	**(19)**
(1) ID	1.0000																		
(2) ID.EA	0.8487 *	1.0000																	
(3) ID.eGHG	0.9542 *	0.6954 *	1.0000																
(4) BoPFS.IFRS	−0.0898 **	−0.1404 *	−0.0970 **	1.0000															
(5) Size	−0.0179	−0.0335	−0.0048	0.2434 *	1.0000														
(6) ROA	0.1340 *	0.0957 **	0.1385 *	−0.1406 *	−0.0693	1.0000													
(7) ROE	0.0888 **	0.0226	0.1103 *	−0.0541	−0.0328	0.6971 *	1.0000												
(8) ROS	0.1167 *	0.1427 *	0.1083	−0.2164 *	−0.2445 *	0.1241 *	0.0073	1.0000											
(9) DebtE	−0.0190	−0.1252 *	0.0277	0.0433	0.0876	−0.1314 *	0.0109	−0.1277 *	1.0000										
(10) DebtTA	−0.0791 ***	−0.1915 *	−0.0270	0.0865	0.1095	−0.2044 *	0.0365	−0.1131 *	0.8905 *	1.0000									
(11) Audit	0.1128 *	0.0548	0.1524 *	0.1774 *	0.0851	−0.0851	−0.0568	−0.1774 *	−0.1064	−0.0568	1.0000								
(12) Aviation	−0.0967 **	−0.1461 *	−0.0625	−0.0255	0.0203	−0.0203	−0.0203	0.1827 *	−0.0406	0.1218 ***	0.0558	1.0000							
(13) Cement	−0.0203	0.0085	−0.0439	−0.0174	0.0426	−0.0426	−0.1193 *	−0.1874 *	−0.1193 *	−0.1193 *	0.1456 *	−0.0436	1.0000						
(14) Combustion	−0.1062 **	−0.0894 **	−0.0816 ***	−0.1477 *	0.1045 **	0.0523	0.0470	0.2143 *	−0.0366	−0.0366	−0.2413 *	−0.1103 *	−0.2880 *	1.0000					
(15) PulpPaper	−0.0249	0.0588	−0.0673	0.0269	−0.2984 *	0.0265	−0.0530	0.0199	−0.0663	−0.0928 ***	−0.0644	−0.0618	−0.1613 *	−0.4082 *	1.0000				
(16) P.Electricity	0.0967 **	−0.0482	0.1230 *	−0.1229 *	0.0079	0.0788 ***	0.1891 *	−0.2521 *	0.2757 *	0.2757 *	0.0845 *	−0.0482	−0.1259 *	−0.3186 *	−0.1785 *	1.0000			
(17) OilRef.	0.0640	0.0448	0.0804 ***	0.0773 ***	0.0000	−0.0496	−0.0992 **	0.0000	−0.0496	−0.0496	0.0454	−0.0136	−0.0355	−0.0898 *	−0.0503	−0.0393	1.0000		
(18) Steel	0.0749 ***	0.0694	0.0566	0.2116 *	0.2037 *	−0.1067 **	−0.0097	0.0291	0.0679	0.0388	0.1243 *	−0.0372	−0.0972 *	−0.2459 *	−0.1378 *	−0.1075 *	−0.0303	1.0000	
(19) Glass	0.0912 **	0.1225 *	0.0982 **	0.1918 *	−0.0317	−0.0528	−0.0528	−0.0211	−0.0739 ***	−0.0844 **	0.1127 *	−0.0338	−0.0881 *	−0.2230 *	−0.1249 *	−0.0975 *	−0.0275	−0.0753 **	1.0000

*, **, *** represent significance levels (two-tailed) at 1%, 5% and 10%, respectively.

**Panel B—Spearman’s correlation matrix (Equation (2)).**

**Table:**

	*(1)*	*(2)*	*(3)*	*(4)*	*(5)*	*(6)*	*(7)*	*(8)*	*(9)*	*(10)*	*(11)*	*(12)*	*(13)*	*(14)*	*(15)*	*(16)*	*(17)*	*(18)*	*(19)*	*(20)*	*(21)*	*(22)*	*(23)*	*(24)*
*(1) IFRIC3_cost*	1.0000																							
*(2) GovGrant*	−0.0491	1.0000																						
*(3) NetLiab*	−0.0264	−0.1001 **	1.0000																					
*(4) Invent*	−0.0120	−0.0454	−0.0244	1.0000																				
*(5) ICAC*	−0.0243	−0.0923 **	−0.0497	−0.0225	1.0000																			
*(6) AFRS26*	−0.0264	−0.1001 **	−0.0538	−0.0244	−0.0497	1.0000																		
*(7) NRec*	−0.0238	−0.0903 **	−0.0486	−0.0220	−0.0448	−0.0486	1.0000																	
*(8) NDisc*	−0.0453	−0.1718 *	−0.0924 **	−0.0419	−0.0852 **	−0.0924 **	−0.0833 ***	1.0000																
*(9) BoPFS.IFRS*	0.0835 ***	0.3169 *	0.1704 *	0.0773 ***	−0.0297	−0.3160 *	0.1156 *	0.0253	1.0000															
*(10) Size*	0.0000	0.1703 *	0.0703	−0.1488 *	0.0000	−0.1641 *	−0.1158 *	0.0225	0.2434 *	1.0000														
*(11) ROA*	−0.0689	0.1064 **	−0.0586	0.0496	0.1009 **	−0.1406 *	0.0901 **	−0.0676	−0.1406 *	−0.0693	1.0000													
*(12) ROE*	−0.0689	0.1419 *	0.0586	0.0000	0.0504	−0.1524 *	0.0386	−0.0601	−0.0541	−0.0328	0.6971 *	1.0000												
*(13) ROS*	0.1609 *	−0.0922 **	−0.1406 *	0.0992 **	0.1135 *	−0.0352	−0.1158 *	0.0000	−0.2164 *	−0.2445 *	0.1241 *	0.0073	1.0000											
*(14) DebtE*	0.0689	0.0922 **	0.1641 *	−0.1488 *	−0.0883 **	0.0469	0.0772 ***	−0.1352 *	0.0433	0.0876 **	−0.1314 *	0.0109	−0.1277 *	1.0000										
*(15) DebtTA*	0.0460	0.0709 ***	0.1524 *	−0.1488 *	−0.1135 *	0.0469	0.0515	−0.0375	0.0865 **	0.1095	−0.2044 *	0.0365	−0.1131 *	0.8905 *	1.0000									
*(16) Audit*	0.0491	−0.0621	0.1001 **	0.0454	−0.0793 ***	−0.0594	0.0903 **	−0.3099 *	0.1774 *	0.0851	−0.0851	−0.0568	−0.1774 *	−0.1064	−0.0568	1.0000								
*(17) Aviation*	−0.0147	−0.0558	−0.0300	−0.0136	−0.0277	−0.0300	−0.0270	0.1992 *	−0.0255	0.0203	−0.0203	−0.0203	0.1827 *	−0.0406	0.1218 ***	0.0558	1.0000							
*(18) Cement*	−0.0384	−0.1456 *	−0.0783 ***	−0.0355	−0.0722 ***	0.3321 *	−0.0706 ***	−0.1343 *	−0.0174	0.0426	−0.0426	−0.1193 *	−0.1874 *	−0.1193 *	−0.1193 *	0.1456 *	−0.0436	1.0000						
*(19) Combustion*	−0.0971 **	−0.2565 *	0.1376 *	0.1233 *	0.2507 *	−0.1981 *	−0.1787 *	0.2084 *	−0.1477 *	0.1045 **	0.0523	0.0470	0.2143 *	−0.0366	−0.0366	−0.2413 *	−0.1103 *	−0.2880 *	1.0000					
*(20) PulpPaper*	−0.0544	0.1288 *	−0.1110 *	−0.0503	−0.1023 *	0.0381	0.2974 *	−0.0267	0.0269	−0.2984 *	0.0265	−0.0530	0.0199	−0.0663	−0.0928 ***	−0.0644	−0.0618	−0.1613 *	−0.4082 *	1.0000				
*(21) P.Electricity*	−0.0425	0.1606 *	−0.0866 **	−0.0393	−0.0799 ***	0.0652	0.0886 **	−0.0514	−0.1229 *	0.0079	0.0788 ***	0.1891 *	−0.2521 *	0.2757 *	0.2757 *	0.0845 *	−0.0482	−0.1259 *	−0.3186 *	−0.1785 *	1.0000			
*(22) Oil Refiner*	−0.0120	−0.0454	−0.0244	−0.0111	−0.0225	−0.0244	−0.0220	−0.0419	0.0773 ***	0.0000	−0.0496	−0.0992 **	0.0000	−0.0496	−0.0496	0.0454	−0.0136	−0.0355	−0.0898 *	−0.0503	−0.0393	1.0000		
*(23) Steel*	0.3948 *	−0.1243 *	0.1201 *	−0.0303	−0.0617	−0.0669	−0.0603	−0.1147 *	0.2116 *	0.2037 *	−0.1067 **	−0.0097	0.0291	0.0679	0.0388	0.1243 *	−0.0372	−0.0972 *	−0.2459 *	−0.1378 *	−0.1075 *	−0.0303	1.0000	
*(24) Glass*	−0.0297	0.4618 *	0.0072	−0.0275	−0.0559	−0.0606	−0.0547	−0.1040 **	0.1918 *	−0.0317	−0.0528	−0.0528	−0.0211	−0.0739 ***	−0.0844 **	0.1127 *	−0.0338	−0.0881 *	−0.2230 *	−0.1249 *	−0.0975 *	−0.0275	−0.0753 **	1.0000

*, **, *** represent significance levels (two-tailed) at 1%, 5% and 10%, respectively.

## Appendix D—Statistic Models: the Influence of Accounting Regulations on the Indexes of the Disclosure

See Table 2 for variable definitions.

This table shows the results for Equation (1), described in Section 2.3.3, using heteroscedastic Panel Corrected Standard Errors (PCSE). Model 1 presents the results for the dependent variable that represents the level of transparency of financial reporting on EA and GHG emissions, model 2 presents the results for the dependent variable that represents the level of transparency of financial reporting on EA using Prais–Winsten regression that corrects first order autocorrelation issues and model 3 presents the results for the dependent variable that represents the level of transparency of financial reporting on GHG emissions. ^(1)^ Wald test for time dummies is not significant.

**Table:**

	**(1) ID**		**(2) ID.EA**		**(3) ID.eGHG**	
	***Coef.***	***Std. Err.***	***Coef.***	***Std. Err.***	***Coef.***	***Std. Err.***
**BoPFS.IFRS**	−0.8402 *	0.2541	−0.5416 **	0.2424	−0.3228 **	0.1329
**Size**	0.1711	0.1855	−0.0345	0.1211	0.0975	0.0949
**ROA**	0.0314	0.2605	0.0282	0.0600	0.1219	0.1337
**ROE**	0.3676	0.2508	0.0253	0.0705	0.1755	0.1272
**ROS**	0.5978 *	0.1811	0.1357 ***	0.0751	0.3069 *	0.0872
**DebtE**	1.4218 *	0.4364	0.3590 **	0.1450	0.7047 *	0.2148
**DebtA**	−1.5074 *	0.4473	−0.1524	0.1470	−0.6186 *	0.2204
**Audit**	1.7479 *	0.4013	0.6490 *	0.2499	0.9450 *	0.1938
**_cons**	1.8618 **	0.9113	1.7397 *	0.4320	0.2075	0.4506
**i.Year**	No^(1)^		Yes		Yes	
***N obs/N groups***	548/85		548/85		548/85	
	chi^2^ (8) = 60.36 *		chi^2^ (14) = 35.24 *		chi^2^ (14) = 64.04 *	
***Hausman chi^2^***	26.94 **		33.06 *		31.37 *	
***AR(1)***	0.330		rho=0.835		0.371	
***Wald chi^2^***	1.4 × 10^8^ *		3.8 × 10^5^ *		3.9× 10^5^ *	
***LM chi^2^***	953.52 *		947.92 *		925.01 *	

*, **, *** represent levels of significance (two-tailed) at 1%, 5% and 10%, respectively.

## Appendix E—Logistic Regressions: the Influence of Accounting Regulations on the Disclosure of Accounting Approaches Statistic Models: the Influence of Accounting Regulations on the Indexes of the Disclosure

See Table 2 for variable definitions.

This table shows the results for Equation (2), described in Section 2.3.3, through Firth’s (1993) Penalized Maximum Likelihood Estimation. *, **, *** represent levels of significance of 1%, 5% and 10%, respectively.

**Table:**

	**(1) IFRIC3_cost**		**(2) GovGrant**		**(3) NetLiab**		**(4) Inventory**		**(5) ICAC**		**(6) AFRS26**		**(7) NRec**		**(8) NDiv**	
	***Coef.***	***Std. Err.***	***Coef.***	***Std. Err.***	***Coef.***	***Std. Err.***	***Coef.***	***Std. Err.***	***Coef.***	***Std. Err.***	***Coef.***	***Std. Err.***	***Coef.***	***Std. Err.***	***Coef.***	***Std. Err.***
**BoPFS.IFRS**	−0.8978	2.8800	5.3734 *	1.5752	3.0022 **	1.4854	4.9124 **	2.2842	0.4792	0.4764	−8.4853 *	2.8815	2.5282 *	0.8312	0.7992 **	0.3409
**Size**	−2.8876	1.9260	2.6690 *	0.4795	−0.5220	0.3442	−4.8690 **	2.1321	0.0126	0.3311	1.6844	1.2739	−1.0232 **	0.4004	−0.0830	0.2254
**ROA**	−1.8704	1.5785	0.3796	0.4705	−0.5879	0.5307	1.1698	1.9157	0.5006	0.5279	−0.9742	0.9509	1.3884 **	0.6107	−0.1607	0.3495
**ROE**	0.9363	1.6338	1.2157 **	0.5012	0.5529	0.4257	−1.1375	1.9803	−0.1213	0.4985	−1.2298	0.8326	−0.7003	0.6201	−0.5054	0.3426
**ROS**	3.6618 **	1.6906	0.6545 ***	0.3958	−0.9763 **	0.4223	0.4112	1.0079	0.3548	0.3427	1.0470	1.0798	−1.0024 **	0.4429	−0.5643 **	0.2240
**DebtE**	1.0276	1.8870	0.2645	0.6306	1.3149 **	0.6443	−1.1202	2.8090	0.4784	0.8797	−2.2846	1.6745	2.5496 ***	1.3017	−1.5548 *	0.4578
**DebtA**	0.0293	2.5682	0.1770	0.6407	−0.3339	0.6669	−1.7385	3.9064	−0.9422	0.8947	4.0748 **	2.0740	−1.8880	1.2806	0.6681	0.4773
**Audit**	0.1271	2.4471	−2.2061 *	0.5353	2.5268 ***	1.4688	−1.3267	2.0880	−0.0725	0.4990	−0.0733	1.1126	3.2177 **	1.6331	−2.2944 *	0.3548
**Aviation**	−4.7746	3.4028	−6.4056 *	2.0933	1.3466	1.9044	5.0777	5.1772	2.0857	2.1055	−7.1057 ***	4.2955	3.3988	2.3195	5.8969 *	1.7164
**Cement**	−4.4409	4.4055	−7.8923 *	1.6211	−1.7274	1.6184	3.0690	2.9951	−0.1952	2.0264	−1.4140	2.4352	−0.2303	2.0437	−0.6079	2.0258
**Combustion**	−4.2134	3.2466	−6.8711 *	0.9024	1.0934	0.7474	2.1054	2.1110	2.2567	1.4638	−12.8288 **	5.4975	−1.2894	2.0162	2.9376 **	1.4486
**PulpPaper**	−5.4676 ***	3.3006	−2.9223 *	0.6310	−2.4641	1.6056	−0.8796	2.3527	−0.9251	2.0180	−3.3876	2.4631	3.0432 **	1.4676	2.1672	1.4697
**P.Electricity**	−5.0719 ***	2.8624	−2.1843 *	0.8293	−2.3606	1.6142	1.7172	2.9785	0.0393	2.0513	−4.8502	3.2045	2.0424	1.5550	2.7249 ***	1.5182
**Oil Ref.**	2.3537	2.2461	−3.7216 **	1.5504	0.8134	1.6637	4.2271	3.1434	1.6912	2.0525	0.9334	2.0857	1.9094	2.1255	1.4649	2.0486
**Steel**	2.1221	2.3374	−9.0969 *	1.7550	1.0417	0.8914	3.5759	3.2872	0.0125	2.0271	−1.8213	2.4410	0.3770	2.0392	0.2953	2.0248
**Glass**	--	--	--	--	--	--	--	--	--	--	--	--	--	--	--	--
**cons**	−5.9999	5.5174	−11.7422 *	2.6302	−7.3692 *	2.6402	2.2574	5.0230	−5.3300 **	2.2466	−1.0186	3.9577	−8.8620 *	2.9971	1.1811	1.7927
**i.Ano**	Não		Não		Não		Não		Não		Não		Não		Não	
**N**	548		548		548		548		548		548		548		548	
**chi^2^ (15)**	17.53		17.44 *		37.55 *		12.46		24.14 ***		31.40 *		40.44 *		77.50 *	
**Penalized Log likelihood**	−9.9687		−69.9257		−60.2749		−9.8626		−69.0688		−28.437		−42.937		−136.3877	
